# Supplementary material for: Immunogenicity and Safety of SARS-CoV-2 Protein Subunit Recombinant Vaccine (IndoVac®) as a Booster Dose against COVID-19 in Indonesian Adults
Source: Vaccines (Basel). 2024 May 14;12(5):540. doi: 10.3390/vaccines12050540 (PMC11125677; doi:10.3390/vaccines12050540)
Supplement: Supplementary file 1 [file vaccines-12-00540-s001.zip › vaccines-2978595-supplementary.pdf]

Supplementary Materials

Table S1. Demographic characteristics**.**

	CoronaVac® group			ChAdOx1 group			BNT162b2 group		
**Parameters**	**IndoVac® vaccine (n = 150)**	**BNT162b2 (n = 150)**	**Total (N =300)**	**IndoVac® vaccine (n = 150)**	**BNT162b2 vaccine (n = 150)**	**Total (N = 300)**	**IndoVac® vaccine (n = 49)**	**BNT162b2-vaccine (n = 47)**	**Total (N = 96)**
Mean age [years] (SD)	38.9 (12.9)	36.9 (13.9)	37.9 (13.4)	36.88 (12.24)	37.54 (12.45)	37.21 (12.33)	37.16 (12.00)	35.64 (11.17)	36.42 (11.56)
Mean height [cm] (SD)	156.4 (9.21)	157.8 (9.2)	157.1 (9.2)	158.03 (9.99)	157.01 (8.92)	157.52 (9.47)	155.75 (9.08)	156.02 (8.67)	155.88 (8.84)
Mean weight [kg] (SD)	61.0 (12.9)	61.85 (13.9)	61.4 (13.4)	63.11 (12.81)	62.59 (13.38)	62.85 (13.08)	57.07 (12.20)	63.13 (16.82)	60.04 (14.88)
BMI (kg/m^2)	24.9 (5.0)	24.7 (4.7)	24.8 (4.8)	25.31 (4.82)	25.46 (5.35)	25.38 (5.08)	23.68 (5.50)	25.96 (6.51)	24.79 (6.09)
Sex, n(%)									
Male	53 (35.33%)	59 (39.33%)	112 (37.33%)	70 (46.67%)	69 (46.00%)	139 (46.33%)	17 (34.69%)	20 (42.55%)	37 (38.54%)
Female	97 (64.67%)	91 (60.67%)	188 (62.67%)	80 (53.33%)	81 (54.00%)	161 (53.67%)	32 (65.31%)	27 (57.45%)	59 (61.46%)
Previous education, n(%)									
Primary school (some)	5 (3.33%)	1 (0.67%)	6 (2.00%)	2 (1.33%)	4 (2.67%)	6 (2.00%)	0 (0.00%)	1 (2.13%)	1 (1.04%)
Primary school (complete)	16 (10.67%)	15 (10.00%)	31 (10.33%)	22 (14.67%)	24 (16.00%)	46 (15.33%)	6 (12.24%)	4 (8.51%)	10 (10.42%)
Junior high school	28 (18.67%)	29 (19.33%)	57 (19.00%)	25 (16.67%)	34 (22.67%)	59 (19.67%)	10 (20.41%)	13 (27.66%)	23 (23.96%)

Parameters	CoronaVac® group: IndoVac® vaccine (n = 150)	CoronaVac® group: BNT162b2 (n = 150)	CoronaVac® group: Total (N =300)	ChAdOx1 group: IndoVac® vaccine (n = 150)	ChAdOx1 group: BNT162b2 vaccine (n = 150)	ChAdOx1 group: Total (N = 300)	BNT162b2 group: IndoVac® vaccine (n = 49)	BNT162b2 group: BNT162b2-vaccine (n = 47)	BNT162b2 group: Total (N = 96)
Senior high school	78 (52.00%)	88 (58.67%)	166 (55.33%)	83 (55.33%)	77 (51.33%)	160 (53.33%)	27 (55.10%)	23 (48.94%)	50 (52.08%)
College or university (diploma)	8 (5.33%)	8 (5.33%)	16 (5.33%)	4 (2.67%)	6 (4.00%)	10 (3.33%)	2 (4.08%)	2 (4.26%)	4 (4.17%)
College or university (bachelor)	11 (7.33%)	7 (4.67%)	18 (6.00%)	12 (8.00%)	4 (2.67%)	16 (5.33%)	4 (8.16%)	4 (8.51%)	8 (8.33%)
Master's or doctoral degree	2 (1.33%)	2 (1.33%)	4 (1.33%)	0 (0.00%)	0 (0.00%)	0 0.00%)	0 (0.00%)	0 (0.00%)	0 (0.00%)
NA	2 (1.33%)	0 (0.00%)	2 (0.67%)	2 (1.33%)	1 (0.67%)	3 (1.00%)	0 (0.00%)	0 (0.00%)	0 (0.00%)
Employment, n(%)									
Yes			205 (68.33%)			220 (73.33%)			62 (64.58%)
State employee/military/police officer	1 (0.67%)	1 (0.67%)	2 (0.67%)	0 (0.00%)	0 (0.00%)	0 (0.00%)	0 (0.00%)	1 (2.13%)	1 (1.04%)
Stated-owned employee	19 (12.67%)	30 (20.00%)	49 (16.33%)	48 (32.00%)	42 (28.00%)	90 (30.00%)	15 (30.61%)	17 (36.17%)	32 (33.33%)
Entrepreneur	15 (10.00%)	15 (10.00%)	30 (10.00%)	10 (6.67%)	20 (13.33%)	30 (10.00%)	2 (4.08%)	2 (4.26%)	4 (4.17%)
Labour	5 (3.33%)	2 (1.33%)	7 (2.33%)	15 (10.00%)	11 (7.33%)	26 (8.67%)	2 (4.08%)	2 (4.26%)	4 (4.17%)

	**CoronaVac® group**						**ChAdOx1 group**						**BNT162b2 group**					
**Parameters**	**IndoVac® vaccine (n = 150)**		**BNT162b2 (n = 150)**		**Total (N =300)**		**IndoVac® vaccine (n = 150)**		**BNT162b2 vaccine (n = 150)**		**Total (N = 300)**		**IndoVac® vaccine (n = 49)**		**BNT162b2- vaccine (n = 47)**		**Total (N = 96)**	
Others	60	(40.00%)	57	(38.00%)	117	(39.00%)	39	(26.00%)	35	(23.33%)	74	(24.67%)	12	(24.49%)	9	(19.15%)	21	(21.88%)
No	50	(33.33%)	45	(30.00%)	95	(31.67%)	38	(25.33%)	42	(28.00%)	80	(26.67%)	18	(36.73%)	16	(34.04%)	34	(35.42%)
Ethnicity, n(%)																		
Alor	11	(7.33%)	10	(6.67%)	21	(7.00%)	10	(6.67%)	13	(8.67%)	23	(7.67%)	0	(0.00%)	1	(2.13%)	1	(1.04%)
Balinese	40	(26.67%)	39	(26.00%)	79	(26.33%)	71	(47.33%)	69	(46.00%)	140	(46.67%)	1	(2.04%)	2	(4.26%)	3	(3.13%)
Batak	2	(1.33%)	1	(0.67%)	3	(1.00%)	0	(0.00%)	0	(0.00%)	0		0	(0.00%)	0	(0.00%)	0	0.00%)
Betawi	1	(0.67%)	0	(0.00%)	1	(0.33%)	0	(0.00%)	0	(0.00%)	0		0	(0.00%)	1	(2.13%)	1	(1.04%)
Buton	0	(0.00%)	1	(0.67%)	1	(0.33%)	0	(0.00%)	0	(0.00%)	0		0	(0.00%)	0	(0.00%)	0	0.00%)
Javanese	20	(13.33%)	23	(15.33%)	43	(14.33%)	19	(12.67%)	19	(12.67%)	38	(12.67%)	8	(16.33%)	12	(25.53%)	20	(20.83%)
Lewae	0	(0.00%)	0	(0.00%)	0	0	(0.00%)	0	(0.00%)	0	0		1	(2.04%)	0	(0.00%)	1	(1.04%)
Malay	0	(0.00%)	1	(0.67%)	1	(0.33%)	0	(0.00%)	1	(0.67%)	1	(0.33%)	0	(0.00%)	0	(0.00%)	0	0
Minangkabau	3	(2.00%)	1	(0.67%)	4	(1.33%)	1	(0.67%)	0	(0.00%)	1	(0.33%)	0	(0.00%)	0	(0.00%)	0	0

	CoronaVac® group			ChAdOx1 group			BNT162b2 group		
Parameters	IndoVac® vaccine (n = 150)	BNT162b2 (n = 150)	Total (N =300)	IndoVac® vaccine (n = 150)	BNT162b2 vaccine (n = 150)	Total (N = 300)	IndoVac® vaccine (n = 49)	BNT162b2- vaccine (n = 47)	Total (N = 96)
Sasak	0 (0.00%)	2 (1.33%)	2 (0.67%)	1 (0.67%)	1 (0.67%)	2 (0.67%)	0 (0.00%)	0 (0.00%)	0 0
Sundanese	73 (48.67%)	72 (48.00%)	145 (48.33%)	48 (32.00%)	47 (31.33%)	95 (31.67%)	39 (79.59%)	31 (65.96%)	70 (72.92%)

Table S2. Serological results**.**

		MICRONEUTRALISING									IGG								
		CORONAVAC®			CHADOX1			BNT162B2			CORONAVAC®			CHADOX1			BNT162B2		
Time Point	Parameter	IndoVac® vaccine	BNT162b2 Vaccine	p-Value	IndoVac® vaccine	BNT162b2 Vaccine	p-Value	IndoVac® vaccine	BNT162b2 vaccine	p-Value	IndoVac® vaccine	BNT162b2 vaccine	p-Value	IndoVac® vaccine	BNT162b2 vaccine	p-Value	IndoVac® vaccine	BNT162b2 vaccine	p-Value
Before booster V1	Number of participants	147	147		149	148		49	46		147	147		149	148		49	46	
	Seropositive rate	113 (77%)	121 (82%)	0.247	129 (87%)	126 (85%)	0.721	44 (90%)	40 (87%)	0.666	145 (98%)	146 (99%)	0.562	149 (100%)	148 (100%)	.a	49 (100%)	46 (100%)	.a
14 days after the booster dose V2	Number of participants	147	147		149	148		49	46		147	147	.a	149	148		49	46	
	Seropositive rate	145 (99%)	147 (100%)	0.156	148 (99%)	148 (100%)	0.318	49 (100%)	46 (100%)	.a	100	100		149 (100%)	148 (100%)	.a	49 (100%)	46 (100%)	.a
	Seroconversion 4-fold increase antibody	74 (65%)	98 (81%)	0.007*	70 (54%)	88 (70%)	0.01*	18 (41%)	30 (75%)	0.002*	116 (80%)	126 (86%)	0.151	92 (61%)	108 (72%)	0.039*	30 (61%)	39 (84%)	0.01*
	Seronegative to seropositive	32 (94%)	26 (100%)	0.208	19 (95%)	22 (100%)	0.288	5 (100%)	6 (100%)	.a	2 (100%)	1 (100%)	.a	0	0	0	0	0	0
	Seroresponse rate	102 (69%)	123 (84%)	0.004*	85 (57%)	110 (74%)	0.002*	22 (45%)	35 (76%)	0.002*	118 (80%)	127 (86%)	0.159	92 (61%)	108 (72%)	0.039*	30 (61%)	39 (84%)	0.01*
28 days after	Number of participants	146	146		149	148		49	46		146	146		149	148		49	46	

**the booster dose V2**																			
	**Seropositive rate**	144 (99%)	146 (100%)	0.156	147 (99%)	148 (100%)	0.157	49 (100%)	46 (100%)	.a	146 (100%)	146 (100%)	.a	149 (100%)	148 (100%)	.a	49 (100%)	46 (100%)	.a
	**Seroconversion 4-fold increase antibody**	80 (71%)	93 (77%)	0.289	64 (50%)	75 (59%)	0.112	23 (52%)	28 (70%)	0.097	112 (77%)	114 (78%)	0.862	92 (61%)	96 (64%)	0.577	30 (61%)	33 (71%)	0.279
	**Seronegative to seropositive**	32 (94%)	26 (100%)	0.208	18 (90%)	22 (100%)	0.129	5 (100%)	6 (100%)	.a	2 (100%)	1 (100%)	.a	0	0	0	0	0	0
	**Seroresponse rate**	109 (75%)	117 (80%)	0.263	77 (52%)	96 (65%)	0.021*	27 (55%)	33 (72%)	0.093	114 (78%)	115 (79%)	0.887	92 (61%)	96 (64%)	0.577	30 (61%)	33 (71%)	0.279
**90 days after the booster dose V3**	**Number of participants**	145	147		146	146		49	45		145	147		146	146		49	45	
	**Seropositive rate**	144 (99%)	145 (99%)	0.570	144 (99%)	146 (100%)	0.156	49 (100%)	44 (98%)	0.294	145 (100%)	147(100%)	<0.001*	146 (100%)	146 (100%)	.a	49 (100%)	45 (100%)	.a
	**Seroconversion 4-fold increase antibody**	53 (48%)	65 (54%)	0.363	67 (53%)	56 (45%)	0.205	19 (43%)	20 (51%)	0.461	100 (70%)	69 (47%)	<0.001*	75 (51%)	54 (37%)	0.013*	22 (45%)	20 (44%)	0.965
	**Seronegative to seropositive**	33 (97%)	24 (92%)	0.403	18 (90%)	22 (100%)	0.129	5 (100%)	5 (83%)	0.338	2 (100%)	1 (100%)	.a	0	0	0	0	0	0
	**Seroresponse rate**	80 (55%)	85 (58%)	0.648	85 (58%)	78 (53%)	0.409	24 (49%)	23 (51%)	0.836	102 (71%)	70 (47%)	<0.001*	75 (51%)	54 (37%)	0.013*	22 (45%)	20 (44%)	0.965
**180 days after the booster dose V4**	**Number of participants**	141	145		145	145		48	45		141	146		145	145		48	45	
	**Seropositive rate**	140 (99%)	144 (99%)	0.984	143 (98%)	144 (99%)	0.562	48 (100%)	43 (95.56%	0.140	141 (100%)	146 (100%)	.a	145 (100%)	145 (100%)	.a	48 (100%)	45 (100%)	.a

Table S3. Geometric mean titre results.

		MICRONEUTRALISING									IGG								
		CORONAVAC®®			CHADOX1			BNT162B2			CORONAVAC®®			CHADOX1			BNT162B2		
Time point	Parameter	IndoVac® vaccine	BNT162b2 vaccine	p-Value	IndoVac® vaccine	BNT162b2 vaccine	p-Value	IndoVac® vaccine	BNT162b2 vaccine	p-Value	IndoVac® vaccine	BNT162b2 vaccine	p-Value	IndoVac® vaccine	BNT162b2 vaccine	p-Value	IndoVac® vaccine	BNT162b2 vaccine	p-Value
Before booster V1	Number of participants	147	147		149	148		49	46		147	147		149	148		49	46	
	GMT (95%CI)	147.52 (117.84–184.66)	151.75 (121.17–190.04)	0.925	251.46 (195.53–323.38)	250.80 (193.44–325.18)	0.877	237.49 (161.61–348.99)	254.56 (164.19–394.66)	0.639	1874.67 (1535.96–2288.064)	1875.98 1547.907–2273.596	0.548	2882.57 (2420.744–3432.498)	2791.88 2345.445–3323.284	0.849	3054.24 (2313.516–4032.119)	3521.95 (2540.014–4883.475)	0.356
	Median	130.15	184.13		260.42	184.13		260.42	314.34		274.80	243.32		2863.90	2793.70		2896.20	3698.20	
	GMR (95%CI)	0.97 (0.71– 1.33)			1.00 (0.69–1.44)			0.93 (0.52–1.66)			1.00 0.758–1.316			1.03 (0.807– 1.319)			0.87 (0.568–1.321)		
14 days after the booster dose V2	Number of participants	147	147		149	148		49	46		147	147		149	148		49	46	
	GMT (95%CI)	1266.69 (1014.73–1581.21)	1982.51 (1659.79–2367.96)	0.002*	1320.43 (1068.11–1632.35)	2142.50 (1830.21–2508.06)	0.002*	1012.50 (710.50–1442.85)	2332.31 (1706.79–3187.06)	<0.001*	19838.60 (17326.833–22714.492)	24525.31 (22519.831–26709.377)	0.232	16329.26 (14428.087–18480.947)	21992.70 20092.897–24072.124	0.001*	16877.87 (13795.343–20649.178)	31913.82 (28586.188–35628.811)	<0.001*
	Median	1041.57	2083.14		1473.00	2083.14		736.50	2946.00		27756.40	28821.60		17259.30	23597.20		18724.10	40000.00	
	GMR (95%CI)	0.64 (0.48 - 0.85)			0.62 (0.47 - 0.80)			0.43 (0.27 - 0.69)			0.81 0.689–0.948			0.74 (0.637–0.865)			0.53 (0.419–0.666)		
14 Days After Booster Dose V2/ Baseline V1	GM Fold (95% CI)	8.59 (6.79–10.85)	13.06 (10.54–16.19)	0.003*	5.25 (4.25–6.49)	8.54 (6.87–10.62)	0.002*	4.26 (3.11–5.84)	9.16 (6.42–13.07)	0.001*	10.58 (8.696–15.7)	13.07 (10.886–15.7)	0.06	5.66 (4.846–6.621)	7.88 (6.655–9.322)	0.017*	5.53 (4.112–7.425)	9.06 (6.625–12.392)	0.01*
	p-Value	<0.001*	<0.001*		<0.001*	<0.001*		<0.001*	<0.001*		<0.001*	<0.001*		<0.001*	<0.001*		<0.001*	<0.001*	
28 Days After Booster Done V2	Number of participants	146	146		149	148		49	46		146	146		149	148		49	46	
	GMT (95%CI)	1455.61 (1178.34–1798.13)	1926.17 (1612.69–2300.58)	0.042*	1039.13 (854.04–1264.33)	1445.64 (1242.48–1682.01)	0.016*	1019.69 (735.06–1414.51)	1661.66 (1250.91–2207.26)	0.006*	18756.85 (16508.38–21311.553)	17930.15 (16151.925–19904.141)	0.176	15054.26 (13412.56–16896.89)	16395.83 (14826.32–18131.50)	0.399	17442.94 (14540.464–20924.783)	26055.58 (22755.132–29834.727)	0.003*
	Median	1473.00	2083.14		1041.57	1473.00		1041.57	2083.14		23536.65	18952.75		15311.40	18021.25		19193.80	31308.70	
	GMR (95%CI)	0.76 (0.57– 0.99)			0.72 (0.56–59.93)			0.61 (0.39–0.94)			1.05 (0.887–1.232)			0.92 (0.788–1.069)			0.67 (0.533–0.839)		

**28 days after the booster dose V2a/ baseline V1**	**GM Fold (95% CI)**	10.05 (7.94–12.71)	12.59 (10.08–15.72)	0.109	4.13 (3.35–5.09)	5.76 (4.56–7.28)	0.044	4.29 (3.22–5.72)	6.53 (4.62–9.21)	0.043*	10.08 (8.235–12.333)	9.44 (7.891–11.286)	0.784	5.22 (4.472–6.098)	5.87 (4.995–6.904)	0.612	5.71 (4.333–7.527)	7.40 5.463–10.017	0.087
	**p-Value**	<0.001*	<0.001*		<0.001*	<0.001*		<0.001*	<0.001*		<0.001*	<0.001*		<0.001*	<0.001*		<0.001*	<0.001*	
**90 days after the booster dose V3**	**Number of participants**	145	147		146	146		49	45		145	147		146	146		49	45	
	**GMT (95%CI)**	717.36 (594.71–865.31)	665.46 (553.36–800.27)	0.538	1352.34 (1116.23–1638.39)	1115.78 (941.01–1323.01)	0.055	956.81 (698.62–1310.40)	1033.56 (698.52–1529.29)	0.597	12710.00 (11197.778–14426.445)	7994.17 (7059.41–9052.707)	<0.001*	11550.42 (10338.929–12903.858)	8949.60 (7951.291–10073.25)	0.003*	12743.28 (10502.196–15462.594)	13645.72 11050.228–16850.839	0.702
	**Median**	736.50	736.50		1473.00	1041.57		736.50	736.50		14208.70	8311.80		12550.20	9418.50		13791.1	1885.04	
	**GMR (95%CI)**	1.08 (0.83–1.40)			1.21 (0.94–1.56)			0.93 (0.57–1.51)			1.59 (1.332–1.897)			1.29 (1.098–1.516)			0.93 (0.704–1.237)		
**90 days after the booster dose V3/baseline V1**	**GM Fold (95% CI)**	5.02 (3.94–6.39)	4.39 (3.51–5.47)	0.564	5.56 (4.18–7.39)	4.55 (3.48–5.94)	0.324	4.03 (2.89–5.62)	4.29 (3.05–6.01)	0.835	6.87 (5.531–8.524)	4.26 (3.591–5.055)	<0.001*	4.07 (3.377–4.906)	3.23 (2.739–3.805)	0.03*	4.17 (3.196–5.445)	3.96 (2.942–5.316)	0.907
	**p-Value**	<0.001*	<0.001*		<0.001*	<0.001*		<0.001*	<0.001*		<0.001*	<0.001*		<0.001*	<0.001*		<0.001*	<0.001*	
**180 days after the booster dose V4**	**Number of participants**	141	145		145	145		48	45		141	146		145	145		48	45	
	**GMT (95%CI)**	758.54 (637.309–902.827)	589.70 (496.939–699.78)	0.059	533.37 (449.581–632.763)	503.63 (430.948–588.565)	0.437	463.94 (343.812–626.039)	416.53 (276.919–626.518)	0.811	1090.63 954.267–1246.474	629.27 (548.847–721.481)	<0.001*	953.66 (842.865–1079.02)	765.02 (675.518–866.371)	0.008*	1012.94 (807.495–1270.662)	973.59 (767.554–1234.94)	0.890
	**Median**	736.50	736.50		520.73	520.73		520.73	520.73		1052.90	628.07		1002.61	760.08		1002.91	946.43	
